# Age‐related changes of tendon fibril micro‐morphology and gene expression

**DOI:** 10.1111/joa.13125

**Published:** 2019-12-03

**Authors:** Iris Ribitsch, Sinan Gueltekin, Marlies Franziska Keith, Kristina Minichmair, Christian Peham, Florien Jenner, Monika Egerbacher

**Affiliations:** ^1^ Department for Companion Animals and Horses Veterm University Equine Hospital Vetmeduni Vienna Vienna Austria; ^2^ Department of Pathobiology Unit of Histology and Embryology Vetmeduni Vienna Vienna Austria

**Keywords:** age, animal model, decorin, equine, fibril diameter, gene expression, horse, tendon

## Abstract

Aging is hypothesized to be associated with changes in tendon matrix composition which may lead to alteration of tendon material properties and hence propensity to injury. Altered gene expression may offer insights into disease pathophysiology and thus open new perspectives toward designing pathophysiology‐driven therapeutics. Therefore, the current study aimed at identifying naturally occurring differences in tendon micro‐morphology and gene expression of newborn, young and old horses. Age‐related differences in the distribution pattern of tendon fibril thickness and in the expression of the tendon relevant genes *collagen type 1 (Col1), Col3, Col5, tenascin‐C, decorin, tenomodulin, versican, scleraxis* and *cartilage oligomeric matrix protein* were investigated. A qualitative and quantitative gene expression and collagen fibril diameter analysis was performed for the most frequently injured equine tendon, the superficial digital flexor tendon, in comparison with the deep digital flexor tendon. Most analyzed genes (*Col1, Col3, Col5, tenascin‐C, tenomodulin, scleraxis*) were expressed at a higher level in foals (age ≤ 6 months) than in horses of 2.75 years (age at which flexor tendons become mature in structure) and older, *decorin* expression increased with age. Decorin was previously reported to inhibit the lateral fusion of collagen fibrils, causing a thinner fibril diameter with increased decorin concentration. The results of this study suggested that reduction of tendon fibril diameters commonly seen in equine tendons with increasing age might be a natural age‐related phenomenon leading to greater fibril surface areas with increased fibrillar interaction and reduced sliding at the fascicular/fibrillar interface and hence a stiffer interfascicular/interfibrillar matrix. This may be a potential reason for the higher propensity to tendinopathies with increasing age.

## Graphical abstract

TEM pictures and histograms showing collagen fibril distribution changes for the SDFT and DDFT with age. Whereas a Gaussian‐like distribution of fibril diameters was observed in tendons of foals up to 6 months of age, a marked shift towards much thinner fibrils was seen in tendons of horses 2.75 years of age or older.

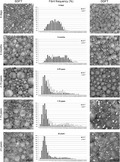

## Introduction

Tendon injuries are the most common musculoskeletal injury in the horse, accounting for up to 46% of all musculoskeletal injuries in athletic horses (Williams et al., 2001; Ely et al., 2004; Kasashima et al., 2004; Lam et al., 2007). The majority of tendon injuries (97–99%) affect forelimb tendons, of which the superficial digital flexor tendon (SDFT) is by far the most commonly affected tendon (75–93% of cases; Ely et al., 2004; Kasashima et al., 2004; Lam et al., 2007; Thorpe et al., 2010). The SDFT, an energy‐storing structure with narrow mechanical safety margins essential for efficient high‐speed locomotion, is the functional and clinical equivalent to the human Achilles tendon (AT; Patterson‐Kane & Rich, 2014). Given the conflicting requirements of elasticity to maximize energy storage and strength to support weight‐bearing, both the SDFT and AT routinely function close to failure levels to store sufficient amounts of energy during athletic activity.

Immature tendons respond to the mechanical forces exerted upon them by altering their structure, composition and mechanical properties – a process called mechanical adaptation (Wang, 2006), whereas mature tendons seem to have limited ability to adapt (Smith et al., 1999). Moreover, aging is hypothesized to play a role in matrix composition changes, which may lead to alteration of the tendon material properties and propensity to injury (Smith et al., 2002). The influence of age and exercise on tendon morphology, collagen fibril distribution, crosslinking and crimp, extracellular matrix composition, molecular and cellular adaptations has been investigated (Michna & Hartmann, 1989; Patterson‐Kane et al., 1997a,b,c, 1998; Smith et al., 1999; Birch et al., 1999a; Edwards et al., 2005; Zhang et al., 2005; Provenzano & Vanderby, 2006; Watanabe et al., 2005, 2007; Sese et al., 2007).

In mature horses, equine tendon collagen fibrils vary in diameter from large to small, resulting in a bimodal diameter distribution (Parry et al., 1978a,b; Patterson‐Kane et al., 1997c) which essentially contributes to the functional properties of tendons and ligaments (Parry et al., 1978a). It has been suggested that the greater surface area of small fibrils may increase the potential for fibrillary interaction, thereby counteracting interfibrillar slippage, whereas larger diameter fibrils have a greater tensile strength (Parry et al., 1978a; Ottani et al., 2001; Birch et al., 2013). Mean collagen fibril diameters decreased in trained horses compared with untrained horses of the same age and the largest fibrils disappeared. The newly formed small‐diameter fibrils are assumed to be subunits of degenerated larger fibrils that had undergone breakdown due to excessive training. Accumulation of micro‐damage may consequently promote the development of clinical tendonitis (Patterson‐Kane et al., 1997c).

Although the effect of exercise on equine tendons has been well described (Cherdchutham et al., 1999), the effect of aging remains poorly characterized. Little is known about natural age‐related changes of tendon specific genes, tendon fibril distribution patterns and their potential interplay. Among other factors, aging is discussed as a potential intrinsic factor likely to be involved in the onset and progression of tendinopathies (Riley, 2004; Thorpe et al., 2013; Docheva et al., 2015; Wezenbeek et al., 2018). It is hypothesized that this may be based on a gradual decrease in mechanical integrity of tendons as individuals age (Riley, 2008). Furthermore, the amount of interfascicular sliding in the SDFT may decrease with increasing age, resulting in a stiffer interfascicular matrix (IFM) and a higher susceptibility to tendinopathies (Thorpe et al., 2013). Although the role of the IFM – which is comprised of collagen type III and proteoglycans and binds the fascicles together – is not yet well defined (Kannus, 2000), it was shown that the IFM becomes stiffer in aged tendons (Thorpe et al., 2013). The reduction in tendon fibril size, which is also associated with aging horses, may increase the potential for fibrillary interaction due to the greater surface area of small fibrils. Together these changes are likely to result in the fascicles/fibrils within the tendon being loaded at an earlier point during tendon extension. The subsequent higher loads experienced by the fascicles during use may predispose these fascicles to damage and lead to increased risks of fatigue‐induced tendon injuries (Thorpe et al., 2013).

The adaptation processes seen during tendon maturation, training and aging are affected by the cells within the tendon tissue (Leadbetter, 1992; Tully et al., 2014; Patterson‐Kane & Rich, 2014). Tenocytes are responsible for synthesis, degradation and maintenance of the extracellular tendon matrix and repair of micro‐damage, and translate mechanical forces into biochemical signals, leading to adaptive physiological or pathological changes in the tissue (Wang, 2006). It seems likely that genes expressed differentially in immature, mature and aging tendons are responsible for a gradual loss of regenerative potential (Smith et al., 1997; Chiquet‐Ehrismann & Tucker, 2004; Halasz et al., 2007; Taylor et al., 2009). The molecular mechanisms governing these age‐related changes are yet to be elucidated. An understanding of these mechanisms would aid in the development of preventative measures and treatments of tendinopathy.

Therefore, the aim of the current study was to identify naturally occurring differences in tendon micro‐morphology and gene expression of horses throughout their lifespan. To that end, age‐related differences in the distribution pattern of tendon fibril thickness and their relation to expression changes of the tendon relevant genes (*Col1, Col3, Col5, tenascin‐C, decorin, tenomodulin, versican, COMP* and *scleraxis*) of collagenous and non‐collagenous matrix components were investigated. Qualitative and quantitative gene expression and collagen fibril diameter analysis were performed, comparing the SDFT with the deep digital flexor tendon (DDFT). The SDFT and DDFT are both flexor tendons and so‐called weight‐bearing tendons (Smith, 2011). The SDFT is the most frequently injured tendon, implicated in up to 93% of soft tissue injuries in Thoroughbred racehorses (Fackelman, 1973; Webbon, 1977; Thorpe et al., 2010) sustaining damage predominantly in the mid‐metacarpal region (Smith, 2011). Injuries to the deep digital flexor tendon (DDFT), on the other hand, typically occur at the phalangeal level and within the digital sheath (Mair & Kinns, 2005; Murray et al., 2006a,b; Smith & Wright, 2006; Dyson & Murray, 2007; Schramme, 2011; Milner et al., 2012). Reports of DDFT lesions at the mid‐metacarpal tensional region are rare (Webbon, 1977; Birch et al., 1999a; Thorpe et al., 2010).

Comparison of immature and mature and aging tendons, as well as comparison of the SDFT with the DDFT, may help identify key factors in developing therapies to reverse tendon degeneration and achieve regeneration. The knowledge gained may benefit both the equine industry and the field of human medicine. Horses are a well‐accepted and well‐established animal model, particularly for musculoskeletal disease (Koch & Betts, 2007; Patterson‐Kane & Rich, 2014).

## Materials and methods

### Sample collection

Sixteen horses (seven female, nine male) with no signs of injuries to the SDFT and DDFT, which were euthanized for reasons unrelated to this study, were included in the study. The horses were between 1 day and 23 years of age. To be able to compare immature and mature tendons, seven horses of the 16 included in the study were foals (age ≤ 6 months) with immature tendons and nine were ≥ 2.75 years old (age at which flexor tendons become mature in structure; Birch et al., 1999b; Smith et al., 2002; Lin et al., 2005).

Tissue collection was performed according to the ‘Good Scientific Practice and Ethics in Science and Research’ regulation implemented at the University of Veterinary Medicine, Vienna. The animal owner’s consent to collect and analyze the samples and to publish resulting data was obtained according to standard procedures approved by the Ethics and Animal Welfare Committee of the University of Veterinary Medicine, Vienna.

As it was our intention to evaluate and describe natural age‐related changes, we only included pleasure riding horses of different breeds with no history of tendon injuries, which had never undergone high‐level athletic training. The foals had also not undergone any training.

Tendon samples were collected from the tendon core of the SDFT and DDFT of one randomly selected front leg at the mid‐metacarpal region, which is the SDFT segment most prone to injury (Fackelman, 1973; Webbon, 1977). All samples were obtained within 3 h postmortem. The samples to be analyzed by transmission electron microscopy (TEM) to evaluate the pattern of tendon fibril thickness, were fixed with 3% glutaraldehyde (C. Roth, Karlsruhe, Germany) and stored at 4 °C until further processing. The samples for gene expression analysis were stored in RNA later (QUIAGEN, Hilden, Germany) at −80 °C until further processing.

### Transmission electron microscopy

The tendon samples were extracted from the glutaraldehyde (3%), subjected to three washing sessions (for 10 min each) in Sörensen phosphate buffer 0.1 m, pH 7.4, and post‐fixed in 1% osmium tetroxide (Electron Microscopy Sciences, Hatfield, PA, USA). After dehydration in a series of ethanol dilutions (70, 80, 96, 100%), samples were embedded in Epon resin (Serva, Mannheim, Germany). Polymerization was allowed for 48 h at 60 °C.

Ultra‐thin (70 nm) transverse sections were cut using an ultramicrotome (Reichert Ultracut S, Leica, Vienna, Austria). The sections were mounted on copper grids (Gröpl, Tulln, Austria) and stained with uranyl acetate (Sigma‐Aldrich, Steinheim, Germany) and lead citrate (Merck, Darmstadt, Germany). Transmission electron micrographs were taken using the EM900 (Zeiss, Oberkochen, Germany) at ×50 000 magnification.

For the evaluation of the fibril diameters and the fibril count, three randomly selected regions of interest (ROI) per ultra‐thin section of tendon were evaluated and five non‐overlapping pictures (*n* = 15 per tendon sample; picture area size 960 × 960 nm) per ROI were taken with a digital Frame‐Transfer‐CCD camera (Tröndle TRS, Moorenweis, Germany) with sis software (ImageSP Professional, TRS). All fibrils in all pictures were measured using ellipse software (Vi‐DiTo, Slovakia). The fibrils were counted and grouped according to their size by applying a 10‐nm scale, ranging from 10 to 360 nm. The number/percentage of fibrils per diameter group was calculated. Results were presented as absolute counts and as percentage using excel 2010.

### Gene expression analysis

#### RNA isolation

Frozen tendon tissues were homogenized in a Biopulverizer (Biospec, USA), which had been pre‐cooled in liquid nitrogen for 1 min. Frozen tendon tissue (10 mg) was placed into one of the designated little pits in the Biopulverizer and pulverized by blowing the hammer onto the pestle designed to match the pits. The tissue powder was transferred to an Eppendorf tube.

For 10 mg of tissue, 500 µL of PureZOL™ (Bio Rad, USA) was added to the tissue powder. The sample was vortexed for 5 min and incubated on ice for 30 min. It was then centrifuged for 10 min at 18 000 *g* at 4 °C. The supernatant was transferred to a new Eppendorf tube and 100 µL of chloroform (Sigma‐Aldrich, Austria) was added. The sample was mixed and incubated at room temperature (RT) for 5 min. The organic and aqueous phases were separated by high‐speed centrifugation (18 000 *g* at 4 °C for 15 min). The aqueous phase was collected into a new Eppendorf tube. Total RNA was recovered by the addition of isopropyl alcohol (Sigma, Austria) and glycerol (Thermo Scientific, USA). The mixture was incubated for 20 min on ice and centrifuged for 30 min at 18 000 *g* at 4 °C. Subsequently, the total RNA pellet was washed twice with 75% ETOH and solubilized in RNase‐free water. Digestion of genomic DNA was carried out with the Ambion™ DNA‐free™ DNA removal kit (Life Technologies, USA) for 30 min at 37 °C.

#### Quantitative PCR (qPCR)

Gene expression analysis was carried out by qPCR for the SDFT and DDFT of all horses included in the study. All primers, except *scleraxis* and *COMP*, were designed using primer3 software. Specificity of the primers was analyzed using the NCBI primer blast tool and *in silico* PCR tool with the UCSC genome browser. The primer design for *scleraxis* and *COMP* was selected according to Taylor et al. (2009). The primer sequences, product length and cycles are shown in Table 1.

**Table 1:** Primer sequences used for qPCR

Gene name	Forward primer sequences	Reverse primer sequences	PCR cycles
*Collagen 1A2*	TCCATCTGGAGAGCCTGGTA	CACCTGGTAGACCACGTTCA	40
*Collagen 3A1*	TGGAGCTCCTGGACTGATAG	CCATTCTTACCAGGCTCACC	40
*Collagen 5A1*	TAGACGTCAACGGCATCATC	TGGTCTGAGACGAAGAGCAG	40
*Tenascin C*	ACCTCAGAGAAGGGCAGACA	CACCGTGAGGTTTTCCAGTT	40
*Decorin*	ACATAACCACCATCCCTCCA	CTCAGGCTAGCTGCATCAAC	40
*Tenomodulin*	CGCCAGACAAGCAAGTGAAG	CATCCAGCATGGGGTCAAAT	40
*Versican*	GTGTGGGCCATGACTATCAG	TCTCATATTGCAGGGTGCTG	40
*Scleraxis B*	TCTGCCTCAGCAACCAGAGA	TCCGAATCGCCGTCTTTC	40
*COMP*	GGTGCGGCTGCTATGGAA	CCAGCTCAGGGCCCTCAT	40
*Gapdh*	GTTTGTGATGGGCGTGAAC	GATGCCAAAGTGGTCATGG	40

RNA (25 ng) from each sample was used for the qPCR reaction. RevTrans QPCR One‐Step EvaGreen kit (Bio&Sell, Germany) was used for the PCR reaction, for cDNA synthesis and subsequently for the qPCR reaction, according to the user manual. The reaction mixtures were incubated for 15 min at 50 °C for cDNA generation, followed by the qPCR reaction: 95 °C for 5 min, 95 °C for 15 s, 55 °C for 20 s and 72 °C for 30 s. For each gene, a reaction mixture without the total RNA template was run as negative control. The transcript data were analyzed using Agilent ariamx 1.1 software (Agilent Technologies, USA). The transcript level for the genes of interest was normalized to the transcriptional level of GAPDH and presented as a relative transcript to *GAPDH*. The choice to use *GAPDH* as housekeeping gene was based on Taylor et al. (2009), who have identified *GAPDH* as a stable reference gene for tendon tissue.

### Statistics

Statistical analysis was performed using SPSS (IBM SPSS statistics, version 24). A Kolmogorov–Smirnov Test for normal distribution was carried out. Correlation between age and fibril diameter, as well as age and fibril count, was determined by Pearson correlation analysis for normally distributed values and by Spearman correlation analysis for values without normal distribution. Overall differences in fibril count and fibril diameters between the SDFT and DDFT were analyzed using a *t*‐test for dependent samples (SDFT and DDFT samples were obtained from the same animals). Differences in fibril counts and fibril diameters between different ages were analyzed using a *t*‐test for independent samples.

## Results

### Transmission electron microscopy

#### Evaluation of fibril diameters, fibril counts and their distribution over age

**Figure 1:**
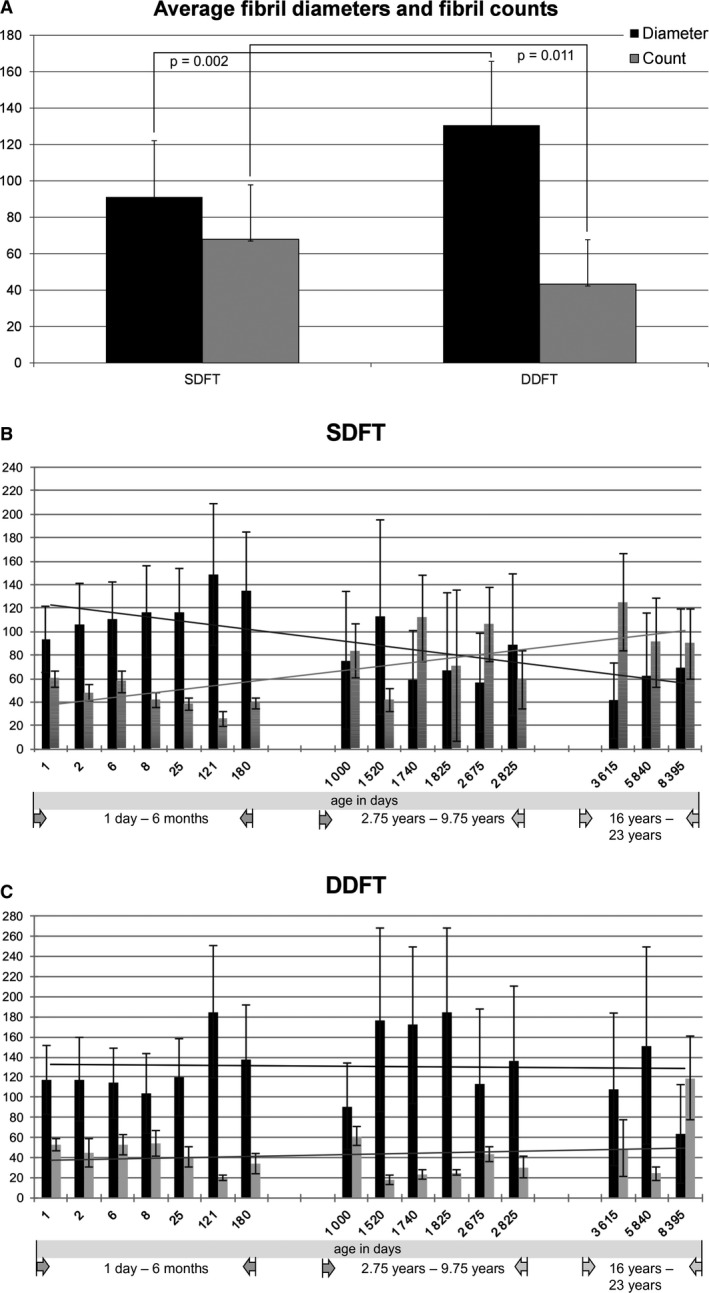
Average fibril diameters (black) and fibril counts (gray) of the SDFT and DDFT (A). The average collagen fibril diameter from all horses was significantly thicker for the DDFT than for the SDFT (*P* = 0.002) (A). The average fibril count (= average number of fibrils counted in 15 non‐overlapping areas per tendon ultra‐thin section) was higher for the SDFT (*P* = 0.011) (A). Error bars indicate standard deviations. Overview of fibril diameters (black) and fibril counts (gray) at different ages (B and C) illustrating the increase of fibril numbers with a concomitant decrease of fibril diameters for the SDFT (B) but not the DDFT (C). Trend lines emphasize these findings (black = fibril diameters, gray = fibril counts). Correlation of SDFT fibril diameters with age: *P* = 0.006, *r* = −0.658; correlation of SDFT fibril counts with age: *P* = 0.009, *r* = 0.627; correlation of DDFT fibril diameters with age: *P* = 0.991, *r* = −0.003; correlation of DDFT fibril counts with age: *P* = 0.499, *r* = −0.182.

The average collagen fibril diameter calculated from all donors was significantly thicker for the DDFT than for the SDFT (DDFT: 130.57 nm, SD: 35.14; SDFT: 91.14 nm, SD: 30.95; *P* = 0.002). Detailed results are depicted in Fig. 1A. Conversely, the average fibril count (= number of fibrils per 960 × 960 nm picture area) was higher for the SDFT (SDFT: 67.94, SD: 29.88; DDFT: 43.26, SD: 24.47; *P* = 0.011; Fig. 1A).

When looking at the overall fibril distribution at different ages, the fibril thickness of the SDFT (but not the DDFT) diminished compared with the fibril count with increasing age (correlation of SDFT fibril diameters with age: *P* = 0.006, *r *= −0.658; correlation of SDFT fibril counts with age: *P* = 0.009, *r* = 0.627; correlation of DDFT fibril diameters with age: *P* = 0.991, *r *= −0.003; correlation of DDFT fibril counts with age: *P* = 0.499, *r *= −0.182), as illustrated in Fig. 1B,C. The detailed values per horse are given in Table 2.

**Table 2:** Horses included into the study and average SDFT and DDFT fibril diameters and counts

Age	Sex	Breed	Average fibril diameter SDFT (in nm)	Average fibril count SDFT	Average fibril diameter DDFT (in nm)	Average fibril count DDFT
1 day	Female	Warmblood	93.57 (± 28.53)	59.8 (± 6.81)	117.26 (± 34.42)	53.06 (± 5.89)
2 days	Female	Arabian	105.53 (± 36.02)	47.53 (± 6.96)	117.81 (± 41.88)	45.07 (± 14.18)
6 days	Male	Welsh Pony	110.55 (± 31.53)	57.27 (± 9.45)	114.08 (± 35.13)	52.83 (± 10.33)
8 days	Female	Warmblood	116.48 (± 39.05)	41.93 (± 6.32)	103.99 (± 39.74)	54.67 (± 12.79)
25 days	Female	Warmblood	116.05 (± 37.86)	38.27 (± 5.57)	119.24 (± 38.29)	40.45 (± 10.42)
4.5 months	Male	Noriker	148.85 (± 60.34)	25.53 (± 6.14)	184.95 (± 66.06)	19.67 (± 2.49)
6 months	Male	Quarter horse	134.44 (± 50.67)	38.93 (± 4.55)	136.82 (± 55.50)	33.77 (± 9.75)
2.75 years	Male	Lusitano	75.41 (± 58.59)	83.53 (± 23.36)	90.84 (± 43.40)	61.06 (± 9.21)
4.5 years	Male	Haflinger	112.66 (± 82.79)	42 (± 10.06)	176.64 (± 91.51)	17.56 (± 4.64)
5 years	Male	Islandic horse	59.34 (± 41.73)	111.4 (± 37.12)	171.98 (± 77.98)	23.46 (± 4.68)
7.25 years	Male	Paint horse	67.46 (± 65.4)	70.73 (± 64.65)	184.29 (± 84.32)	25.13 (± 3.18)
7.75 years	Male	Warmblood	56.47 (± 41.66)	106.3 (± 31.83)	113.12 (± 74.74)	42.77 (± 7.46)
9.75 years	Female	Haflinger	89.1 (± 60.30)	58.87 (± 24.76)	135.43 (± 74.77)	30.22 (± 10.46)
16 years	Male	Noriker	41.12 (± 32.28)	125 (± 41.66)	108.06 (± 75.6)	49.26 (± 28.03)
20 years	Female	Warmblood	62.4 (± 52.97)	90.53 (± 37.99)	151.28 (± 97.79)	24.13 (± 6.87)
23 years	Female	Welsh Pony	68.85 (± 50.88)	89.4 (± 30.0)	63.35 (± 48.51)	119 (± 41.3)

For horses between the ages of 0 and 6 months, the SDFT fibril diameters (black bars) were thick at a low fibril count (gray bars) (Fig. 1B). After the age of 6 months, the average fibril diameter diminished compared with the average fibril count (Fig. 1B). In the DDFT, fibril distribution was similar between all age groups (Fig. 1C). No trend toward an age‐related diminishment of average fibril diameters (black bars) compared with the average fibril count (gray bars) was observed (Fig. 1C).

**Figure 2:**
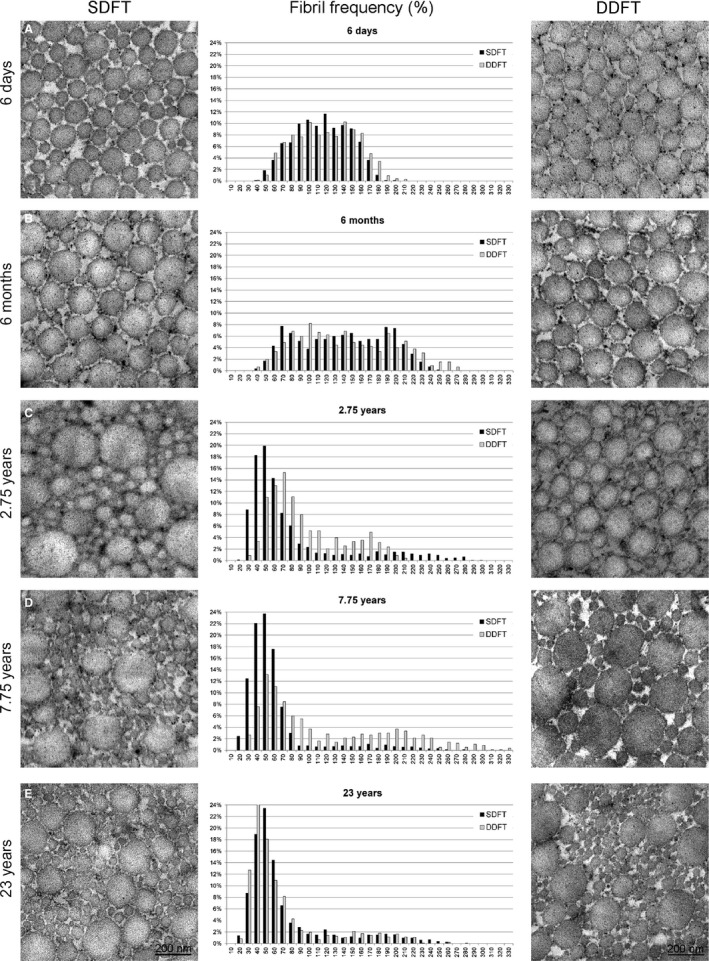
Representative TEM pictures and respective histograms showing collagen fibril distribution changes for the SDFT and DDFT with age. Whereas a Gaussian‐like distribution of fibril diameters was observed in tendons of foals up to 6 months of age, a marked shift towards much thinner fibrils was seen in tendons of horses 2.75 years of age or older.

Both the SDFT and DDFT showed a rather unimodal fibril diameter distribution in foals (up to 6 months) but a wider range of fibril diameters, resulting in a bimodal to trimodal distribution in mature tendons (2.75–23 years; Fig. 2). The thickest (> 250 nm) and thinnest (< 30 nm) fibril diameters were therefore only found in mature tendons (2.75–23 years), with one exception, the 4‐month‐old Noriker foal, for which we found fibrils with diameters greater than 270 nm in both the SDFT and DDFT. Detailed results illustrating fibril diameters and fibril counts at different ages are depicted in Figs 1B,C and 2 and Table 2.

For very young horses (up to 6 months of age) a similar general fibril distribution of the SDFT and DDFT was found (Fig. 2A,B). During the first days of life (1–25 days) the distribution range was narrow (between 25 and 250 nm, with most fibril diameters ranging between 55 and 190 nm) and the overall distribution pattern resembling a Gaussian distribution curve (Fig. 2A,B). At the age of a few months (4 and 6 months) the overall distribution pattern was wider – from 25 up to 315 nm, but still with a Gaussian‐like curve shape and similar between the SDFT and the DDFT (Fig. 2B). From the age of 2.75 years, we found a marked shift of the fibril distribution pattern towards thinner fibrils, the majority of fibrils ranging between 15 and 75 nm for the SDFT and between 25 and 105 nm for the DDFT (Fig. 2C). This shift appeared to be delayed in the DDFT compared with the SDFT. However, the overall distribution range remained the same (between 15 and 275 nm for the SDFT and between 25 and 205 nm for the DDFT; Fig. 2C,D). Interestingly, although the 2‐year‐old horse already displayed a marked shift towards thin fibrils for the SDFT and DDFT – similar to older horses – it did not show widening of the spectrum toward very thick fibrils in the DDFT (Fig. 2C). After the age of 4 years, the fibril diameter distribution range of the DDFT became wider, with a particular increase in thick fibrils (up to 355 nm), but with an overall, almost even distribution of fibril counts up to the age of 7 years. For the SDFT, the overall fibril thickness distribution range remained similar, but with a constant shift towards higher counts of the thin fibrils, which became more and more pronounced with increasing age (majority of fibril diameters ranging between 25 and 70 nm; Fig. 2B–E). For the DDFT, a constant shift towards thinner fibrils was found after the age of 7.75 years (Fig. 2C–E). At the age of 23 years, the distribution curves for the SDFT and DDFT looked the same, with an overall fibril distribution of 15–255 nm. The majority of fibrils ranged between 25 and 65 nm for both the SDFT and DDFT (Fig. 2E).

#### Correlation of age with fibril counts and fibril diameters

Overall, the average fibril diameter of the SDFT decreased with increasing age (*P* = 0.004, *r *= −0.677). Conversely, the fibril count increased with age (*P* = 0.007, *r* = 0.643). The DDFT did not show significant changes in fibril diameters or counts associated with age. Overall trends in fibril diameter and fibril count changes for the SDFT and DDFT are illustrated in Fig. 1B,C, where the fibril counts and diameters for each horse included in the study are plotted.

### Gene expression analysis

**Figure 3:**
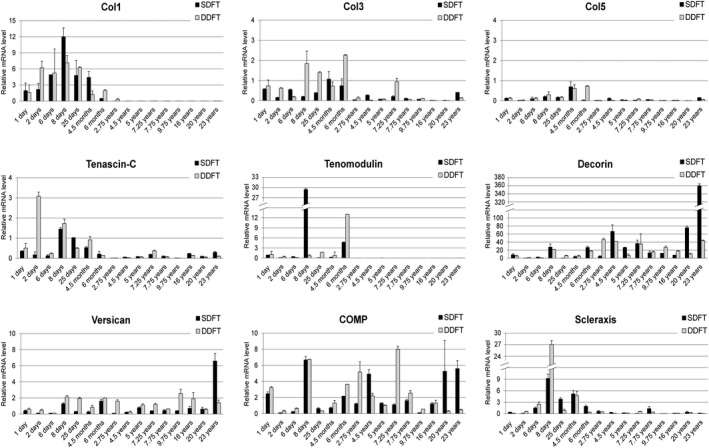
Expression of tendon‐relevant genes for the SDFT (black bars) and DDFT (gray bars). The graphs show the individual expression (log scale) of tendon‐relevant genes (mRNA level relative to GAPDH) for all horses included in the study and illustrates the clear shift in gene expression of *Col1, Col3, Col5, tenascin, tenomodulin* and *scleraxis* (a decrease to almost 0) as well as *decorin* (an increase) after the age of 6 months.

Expression changes of the tendon‐relevant genes *Col1, Col3, Col5, tenascin‐C, decorin, tenomodulin, versican, COMP* and *scleraxis* were investigated for the SDFT and DDFT of all 16 horses included in the study. In accordance with the shift in fibril diameters and fibril counts, there was a clear shift in gene expression of *Col 1, Col 3, Col 5, tenascin, tenomodulin* and *scleraxis* (a decrease to almost 0), as well as *decorin* (an increase) after the age of 6 months. The results of the gene expression analysis are illustrated in Fig. 3.

*Col1* expression was detectable only for horses with an age of 4 years or younger, in which the expression level was overall higher in the DDFT than in the SDFT. For both the SDFT and DDFT, *Col1* expression was highest in the 8‐day‐old foal. In the 2‐year‐old horse, *Col 1* expression decreased to very low levels (SDFT: 0.0006 and DDFT: 0.3). In horses older than 4 years, no expression of *Col 1* was detectable any longer.

*Col3* showed a similar trend as *Col1,* with a more constant and higher expression up to the age of 6 months. After 6 months, the expression levels dropped, except for the 7‐year‐old horse (DDFT) and the 23‐year‐old horse (SDFT). For the DDFT, *Col3* expression levels remained higher.

Additionally, *Col5* expression levels were highest in very young horses. The expression levels dropped after the age of 4 months, particularly for the DDFT.

*Tenascin‐C* expression levels were higher for horses with an age of 4 months and younger, for both the SDFT and DDFT.

*Tenomodulin* expression could only be detected in horses under the age of 6 months. As for *Col1,* the highest expression levels were found for the 8‐day‐old foal (SDFT).

For *COMP,* no age‐related expression pattern could be found – either for the SDFT or for the DDFT. However, again, the 8‐day‐old foal showed the highest expression level for the SDFT.

For *versican,* no age‐related expression pattern could be found – either for the SDFT or for the DDFT.

*Scleraxis* also showed higher expression in horses up to an age of 6 months, with an overall higher expression level in the SDFT than in the DDFT. However, for the 8‐day‐old foal, which showed the highest expression levels for *scleraxis* in both the SDFT and DDFT, particularly high expression was found in the DDFT.

In contrast to all other genes, *decorin* expression was overall higher in horses older than 6 months. Interestingly, among the horses below the age of 6 months, the 8‐day‐old foal again showed the highest expression levels.

#### Correlation of gene expression levels with age, fibril counts and fibril diameters

For the SDFT there was a negative correlation between age and *Col1* (*P* = 0.004, *r *= −0.673), *Col3* (*P* = 0.038, *r *= −0.522) and *tenomodulin* (*P *= < 0.000, *r *= −0.829), and a positive correlation between age and *decorin* (*P* = 0.011, *r* = 0.615). Further fibril diameters correlated positively with the expression of *Col1* (*P* = 0.014, *r* = 0.600), *Col3* (*P* = 0.002, *r* = 0.712), *Col5* (*P* = 0.040, *r* = 0.518), *tenomodulin* (*P* = 0.010, *r* = 0.624) and *scleraxis* (*P* = 0.013, *r* = 0.606), and fibril counts correlated negatively with the expression of *Col1* (*P* = 0.015, *r *= −0.594), *Col3* (*P* = 0.004, *r *= −0.674), *tenomodulin* (*P* = 0.025, *r *= −0.556) and *scleraxis* (*P* = 0.033, *r *= −0.535). The negative correlation between *Col5* and the fibril counts did not reach statistical significance (*P* = 0.060, *r *= −0.479).

For the DDFT there was a negative correlation between age and *Col1* (*P *= < 0.000, *r *= −0.801), *Col3* (*P* = 0.010, *r *= −0.623), *Col5* (*P* = 0.025, *r *= −0.556), *tenascin* (*P* = 0.004, *r *= −0.673), *tenomodulin* (*P* = 0.006, *r *= −0.658) and *scleraxis* (*P* = 0.012, *r *= −0.609), and a positive correlation between age and *decorin* (*P* = 0.015, *r* = 0.593). There was no statistically significant correlation between DDFT fibril diameters or counts with the expression level of the analyzed genes.

#### Differences between immature and mature tendons

As demonstrated in previous studies, the time from birth to approximately 6 months of age is a period of rapid growth and development, particularly of the musculoskeletal tissues. In this period, a large number of dynamic remodeling processes take place which profoundly influence the histological, biochemical and biomechanical characteristics of musculoskeletal tissues (Cherdchutham et al., 1999, 2001; Brama et al., 2000, 2002, van Weeren, 2000).

**Figure 4:**
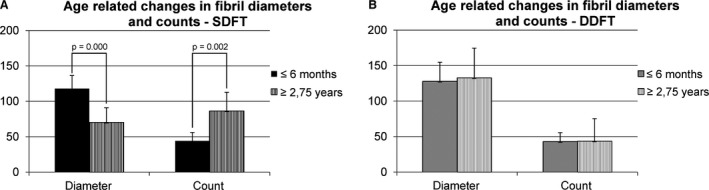
Changes in fibril diameters and counts for the SDFT (A) and DDFT (B) between foals (≤ 6 months) and horses with mature tendons (≥ 2.75 years of age). The average fibril diameter of the SDFT decreased with increasing age (*P* = 0.000). Conversely, the fibril count increased with age (*P* = 0.002) (A). The DDFT did not show significant changes in fibril diameters or counts associated with age (B).

In the current study, the most striking differences for all analyzed parameters (fibril diameters and fibril counts, fibril diameter distributions and gene expression of tendon relevant genes) are between immature (≤ 6 months) and mature tendons (≥ 2.75 years). Between the ages of 6 months and 2.75 years, the fibril thickness of the SDFT (but not the DDFT) diminished in comparison with the fibril count (Fig. 1B,C). The most pronounced changes in gene expression levels occurred between the ages of 6 months and 2.75 years. Expression of *Col1, Col3* and *Col5*, as well as *tenascin C*,* tenomodulin* and *scleraxis* apparently ceased after the age of 6 months, whereas *decorin* expression levels increased (Fig. 3). Since the maturation of foals appears to be a critical time point, the horses were grouped as foals (age ≤ 6 months) and horses with mature tendons (age ≥ 2.75). For the SDFT, a thick average collagen fibril diameter (117.92 nm, SD = 18.42 nm) was demonstrated, with a low average fibril count (44.18, SD = 11.84) in horses up to an age of 6 months. After that age, the average fibril diameter diminished (70.31 nm, SD = 20.64) and the average fibril count increased (86.42, SD = 26.27). The differences between the two groups (below and above the age of 6 months) were statistically significant (*P *> 0.000 for fibril diameters and *P* = 0.002 for fibril counts; Fig. 4A). In contrast, the average fibril diameter (127.73 nm, SD = 27.04 nm) and fibril count (42.8 SD = 12.74) of the DDFT in horses 6 months and younger was similar to that in horses older than 6 months. A slightly higher average fibril diameter of 132.8 nm (SD = 41.85) and a fibril count of 43.6 (SD = 31.63) were detected in the older age group (Fig. 4B). These differences were not statistically significant.

## Discussion

The pathophysiology of tendinopathies is a complex and multifactorial process which is not yet completely understood. It is influenced by many intrinsic and extrinsic factors such as age, genetics, anatomical variants, bodyweight, systemic disease, sporting activities, physical loading, occupation and environmental conditions, resulting in accumulated micro‐damage due to repetitive strain. This is thought to lead to weakening of the collagen crosslinks, the non‐collagenous matrix and the vascular elements (Smith & Webbon, 2005; Abate et al., 2009; Docheva et al., 2015). Unfortunately, due to the hypocellularity and the low metabolic rate of tenocytes, the natural healing ability of tendons is minimal (Riley, 2008). Thus, adult injured tendons fail to regenerate and form scar tissue with significantly inferior biomechanical properties and a propensity to re‐injure (Bruns et al., 2000; Gajhede‐Knudsen et al., 2013). This highlights the importance of efforts to develop new therapies for tendon regeneration (Patruno & Martinello, 2014). However, development of any new regenerative therapies requires a thorough understanding of the physiology and pathophysiology of the tissue of interest (Patruno & Martinello, 2014). Therefore, we carried out an explorative study investigating natural age‐related changes in collagen fibril diameters and gene expression of the equine SDFT and DDFT. Altered gene expression may offer disease‐relevant information, furthering our understanding in order to design pathophysiology‐driven therapeutics. The goal of investigating natural, age‐related changes/adaption processes is reflected in the study design: two crucial aspects were the inclusion criteria for recruited horses and the anatomic location from which tendon samples were obtained. The study population had no history of tendon‐related disease and had never undergone a particularly strenuous exercise program. Furthermore, all samples were taken from the tendon core of the mid‐metacarpal region – the region most frequently injured in SDFTs.

The results of this study show a clear difference in fibril diameter distribution and gene expression between foals (age 1 day–6 months) and horses with mature tendons (age 2.75–23 years). The period from birth to approximately 6 months of age is a time of fast growth and rapid development, particularly of musculoskeletal tissues. It has been demonstrated that during this period a number of dynamic remodeling processes take place which profoundly influence the histological, biochemical and biomechanical characteristics of musculoskeletal tissues. Between the ages of 6 and 11 months, these processes are already occurring at a slower rate. There are indications that most of the final constitution of certain tissue components has already formed before the age of 6 months (Cherdchutham et al., 1999, 2001; Brama et al., 2000, 2002; van Weeren, 2000).

In accordance with Parry et al. (1978a), Parry et al. (1978b) and van Weeren (2000) we found a unimodal fibril diameter distribution in the SDFTs and DDFTs of foals (up to 6 months). In mature horses, a bimodal to trimodal distribution, with a concomitant reduction in average fibril size but an increase in fibril counts with age, were observed. Generally, we found a wider fibril distribution spectrum in mature tendons compared with that in foals (Fig. 2). Nonetheless, mature tendons contained only a few fibrils with very large diameters (250–355 nm), which were not commonly found in foals. The large number of relatively thin fibrils interspersed with very large fibrils found in the adult tendon samples confirms results of previous studies (Patterson‐Kane et al., 1997c; Parry et al., 1978a,b; Edwards et al., 2005). It is not clear, however, what the stimulus for this change in equine tendons is and whether it occurs as part of a natural aging process, as an exercise‐induced phenomena or both. Aging and athletic training are both factors which were reported to result in progressive changes in the nature of the extracellular matrix of tendons (Birch et al., 1999a). In comparison with the SDFT, the DDFT appears to undergo less noteworthy changes of its ultrastructure in response to age (Fig. 1B,C). This is likely due to the comparatively lower functional and biomechanical demands exerted upon the DDFT compared with the SDFT (Patterson‐Kane et al., 1998).

However, the typical fibril distribution pattern appears to vary not only between different ages and tendons, but also between different locations within one tendon. Fibrils in the tendon core tend to have a smaller diameter than fibrils from the tendon periphery (Sese et al., 2007). Also, the number of large diameter fibrils is increased in the distal compared with the proximal and middle third of the SDFT (Watanabe et al., 2005). All samples in the current study were therefore obtained from the same tendon region (core of the mid‐metacarpal region). The shift to smaller collagen fibril diameters with age found in horses is in contrast to previous findings in rats, rabbits and mice, which showed an age‐related increase in fibril diameter (Moore & De Beaux, 1987; Oryan & Shoushtari, 2008; Ansorge et al., 2011; 2012; Dunkman et al., 2013).

There is little doubt that the cells affect adaptive processes in tissues. Therefore, we aimed to provide an overview of age‐related changes in the expression levels of *Col1, Col3, Col5, tenascin‐C, decorin, tenomodulin, versican, COMP* and *scleraxis* for the SDFT and DDFT (Fig. 3). The gross expression levels for the investigated genes were found to be comparable to levels reported by Taylor et al. (2009), confirming the validity of our results. Gene expression analysis revealed that most analyzed genes (*Col1, Col3, Col5, tenascin‐C, tenomodulin, scleraxis*) were expressed at a higher level in foals (< 6 months) than in horses 2.75 years of age (the age at which flexor tendons become mature in structure; Birch et al., 1999b; Smith et al., 2002; Lin et al., 2005) and older.

In this study, the most striking differences for all analyzed parameters (fibril diameters and fibril counts, fibril diameter distributions and gene expression of tendon relevant genes) were found between immature (≤ 6 months) and mature tendons (≥ 2.75 years). This result may be considered complementary to Thorpe et al. (2016) who showed in their study that aging does not result in a decline in cell synthetic activity after the age of 3 years.

The findings by Thorpe et al. (2016) and our study also support the assumption that whereas energy‐storing tendons appear to be able to respond to the mechanical forces during growth, there is no evidence that they can do so after skeletal maturity. Instead, cumulative fatigue damage may cause degeneration, potentially weakening tendons and increasing the risk of tendon injury (Smith et al., 2002). However, in contrast to the other genes, *decorin* expression was considerably higher in mature tendons (≥ 2.75 years) than in the tendons of foals (Fig. 3). Decorin is a small, leucine‐rich proteoglycan combining dermatan sulfate (DS) and chondroitin sulfate (CS), which binds to the surface of collagen fibrils and is involved in their assembly and lateral growth in connective tissue types such as tendons (Vogel & Trotter, 1987; Watanabe et al., 2005). Decorin was repeatedly suggested to inhibit the lateral fusion of collagen fibrils with increased decorin concentration, causing a thinner fibril diameter (Danielson et al., 1997; Watanabe et al., 2005). *In vitro*, small dermatan sulfate proteoglycans were shown to be involved in the formation of significantly thinner collagen fibrils and a retarded rate of fibril diameter increase, indicating an inhibition of lateral aggregation during collagen fibril formation (Vogel & Trotter, 1987).

Genetic evidence that decorin may play a fundamental role in collagen fibrillogenesis was found using mice with a targeted disruption at the *decorin* gene locus. These mice developed fragile skin with reduced tensile strength and abnormal collagen fibrillogenesis associated with a decrease in collagen‐bound proteoglycans in the skin and tendons (Danielson et al., 1997). Moreover, during development of chicken embryos, fibril‐associated decorin was shown to decrease as fibril length and diameter increase (Birk et al., 1995). Watanabe et al. (2005) reported that regional differences in fibril diameter are partly due to fusion of fibrils and the concomitant regional differences in decorin amount and size.

In contrast to all these findings, the presence of a subpopulation of larger diameter fibrils in tendons of aging mice was reported to be associated with continued expression of *decorin* (Ansorge et al., 2012; Dunkman et al., 2013). This discrepancy may be due to a potentially highly variable and locally influenced role of decorin (Reed & Iozzo, 2002).

Previously it was hypothesized that the reduction in fibril diameter in the injury‐prone core region of tendons was an indication of micro‐trauma, with the new small diameter fibrils being subunits of degenerated large fibrils or newly synthesized by stimulated tenocytes (Patterson‐Kane et al., 1997c). This apparently corresponds with the finding of increased levels of chondroitin sulfate equivalent glycosaminoglycans in the central regions of degenerated tendons (Birch et al., 1998). However, results of our current study suggest that changes seen in collagen morphology, particularly of the SDFT (shift towards thinner fibrils) with age, may be related to a natural increase in *decorin* expression and decrease of most other analyzed tendon relevant genes (*Col1, Col3, Col5, tenascin‐C, tenomodulin, scleraxis*) in mature tendons. The described molecular changes may contribute to the formation of thinner collagen fibrils with greater surface area and hence increased fibrillary interaction (Parry et al., 1978a; Ottani et al., 2001; Birch et al., 2013) and increased IFM stiffness due to a reduction in sliding capacity in mature individuals (Thorpe et al., 2013).

Although we intentionally included only pleasure horses with a low likelihood of exercise‐induced micro‐damage and with no history of tendon injuries, the extremely high *decorin* expression detected in the SDFT of the 23‐year‐old horse may indicate subclinical damage which had not been diagnosed. However, even after removing the results of the 23‐year‐old horse from the dataset, the correlation between decorin and age remained statistically significant (*P* = 0.043, *r* = 0.532 without the 23‐year‐old horse vs. *P* = 0.011, *r* = 0.615 with the 23‐year‐old horse), so despite this potentially questionable sample, the trend to higher *decorin* levels with increasing age remains obvious. Our analogous findings of increasing *decorin* gene expression and protein secretion in mature compared with fetal sheep further substantiate our results (I. Ribitsch, S. Gueltekin, F. Jenner, A. Bileck, R. Mayer, C. Gerner, unpublished results).

The less pronounced changes in the DDFT, particularly with regard to fibril thickness and fibril counts compared with the SDFT, may be due to their different function – energy‐storing (SDFT) vs. non‐energy‐storing tendon (DDFT) – which may make the DDFT less prone to injury (Webbon, 1977; Birch et al., 1999a).

Building on these results, it should further be investigated whether *decorin* concentration and/or molecular size could be targeted to treat or prevent micro‐trauma‐induced fibril thinning in mature tendons. All the more so, because our findings are further supported by recent reports linking the occurrence of equine degenerative suspensory ligament desmitis to an abnormal accumulation of proteoglycans and particularly decorin (Young et al., 2018).

The present study has some limitations. In addition to a relatively low sample size, there is a lack of samples between the age of 6 months and 2.75 years. This is due to the fact that all horses included into this study were euthanized for reasons unrelated to this study and no horses of this age group were euthanized during the study period. However, although inclusion of horses in this age range, in which significant growth occurs, would have been desirable, indications that most of the final constitution of certain tissue components has already formed before the age of 6 months (Cherdchutham et al., 1999, 2001; Brama et al., 2000, 2002; van Weeren, 2000), allow for firm conclusions despite the missing samples between 6 months and 2.75 years.

Furthermore, the study was performed on a limited set of genes which are known to play an important role in tendon biology. Future studies should probably include a broader set of genes, e.g. using whole genome microarrays or next generation sequencing. Additional analyses of the age‐specific protein synthesis, rather than changes of the intrinsic cellular function based merely on gene expression, may offer valuable information about the molecular biology dictating tendon development, maturation and aging. This was also suggested by Thorpe et al. (2016) who found that an important mechanism for age‐related tendon deterioration might be connected with protein post‐translational modifications.

Including horses of different breeds may have influenced the results. However, as the overall tendon biology is the same for horses of different breeds, any potential impact may be considered minor.

In summary, our results confirm morphological findings of previous studies describing an age‐related shift toward thinner fibril diameters in equine tendons, particularly the SDFT, and offer a first attempt to unravel the molecular mechanisms governing these age‐related changes. Based on the concomitant increase of *decorin* expression and decrease of other tendon extracellular matrix genes found in this study, which was carried out on horses with no history of excessive training or tendon injuries, we suggest that the reduction of fibril diameter may be a natural age‐related process. The resulting greater fibril surface areas and increased fibrillary interaction paired with reduced sliding at the fascicular interface and hence a stiffer IFM, may explain the higher susceptibility to tendinopathies with increasing age. Further research will be necessary to understand fully the molecular background of age‐related tendon weakening in order to aid in the development of preventative measures and treatments of tendinopathy. Due to the similarity of the equine SDFT and the human AT, results of our study may be relevant not only for equine but also for human medicine.

## Conflict of interest

The authors declare that they have no conflict of interests.

## Author contributions

I.R. designed the study, collected the samples, interpreted the data and wrote the manuscript. S.G. performed the qPCR analysis and interpreted the data. M.F.K. performed TEM of the SDFT and interpreted the data, K.M. performed TEM of the DDFT and interpreted the data, C.P. did the statistical analysis, F.J. substantively revised the manuscript, M.E. designed the study, interpreted the data and substantively revised the manuscript. All authors read and approved the final manuscript.

## Data Availability

All data generated or analyzed during this study are included in this article.
